# Supplementary material for: Impact of Whole Genome Doubling on Detection of Circulating Tumor DNA in Colorectal Cancer
Source: Cancers (Basel). 2023 Feb 10;15(4):1136. doi: 10.3390/cancers15041136 (PMC9954709; doi:10.3390/cancers15041136)
Supplement: Supplementary file 1 [file cancers-15-01136-s001.zip › Supplementary Figures.pdf]

Supplementary Figures

Figure S1

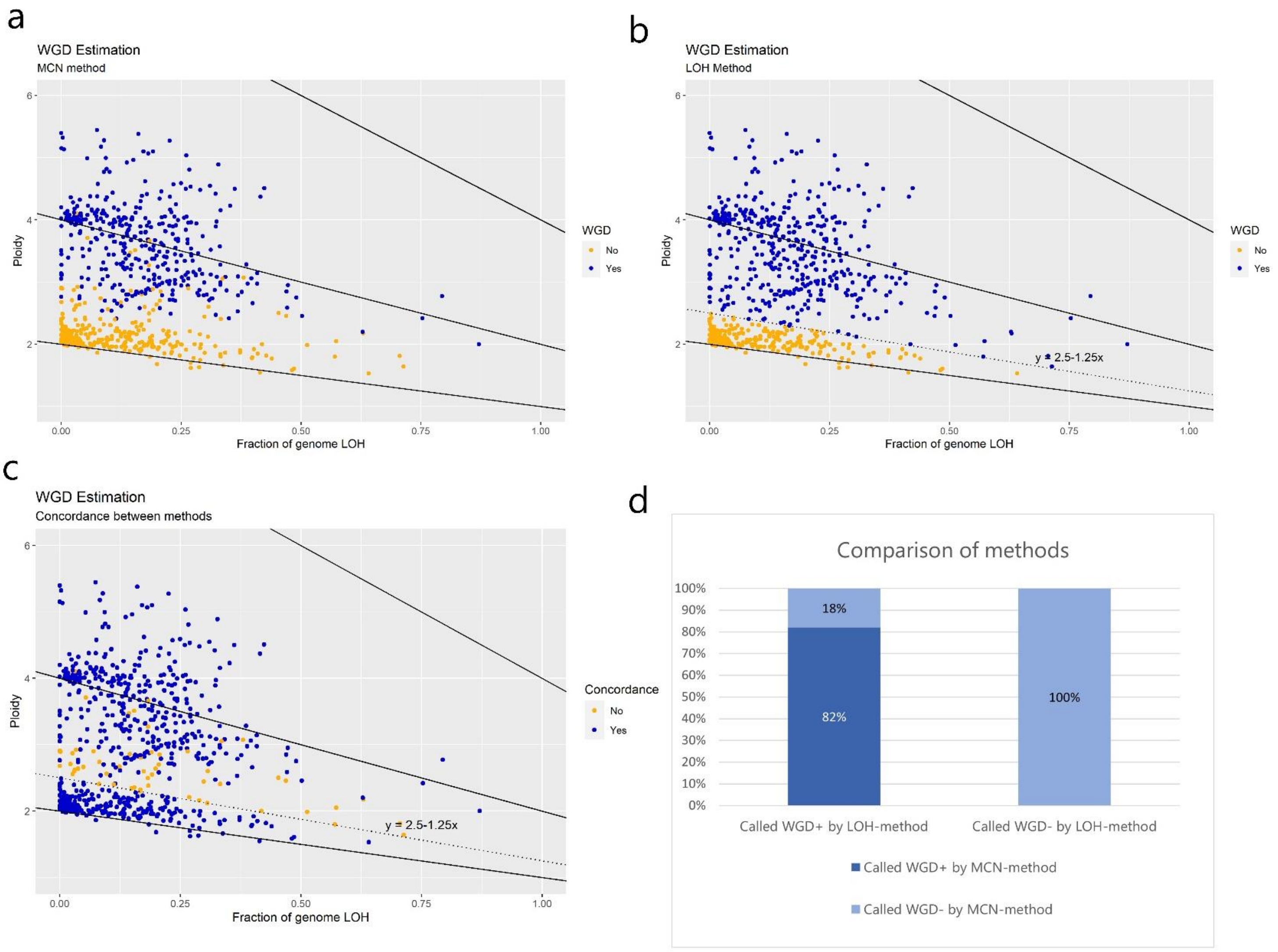

Figure S1: Overview of WGD estimation methods. ***a****: WGD-estimation using LOH-method. Solid lines indicate the expected ploidy of a diploid, tetraploid and octoploid cell with increasing fractions of the genome having undergone LOH. Dotted line indicates the cutoff-line between WGD+ and WGD-.* ***b****: For comparison, same plot as a) but colored according to WGD-estimation using the MCN method utilized in this paper.* ***c****: Concordance between the LOH and MCN methods. Overall concordance = 92%.* ***d****: Proportions of LOH-calls being called WGD+ or WGD- by MCN method. Cohen's Kappa = 0.83.*

Figure S2

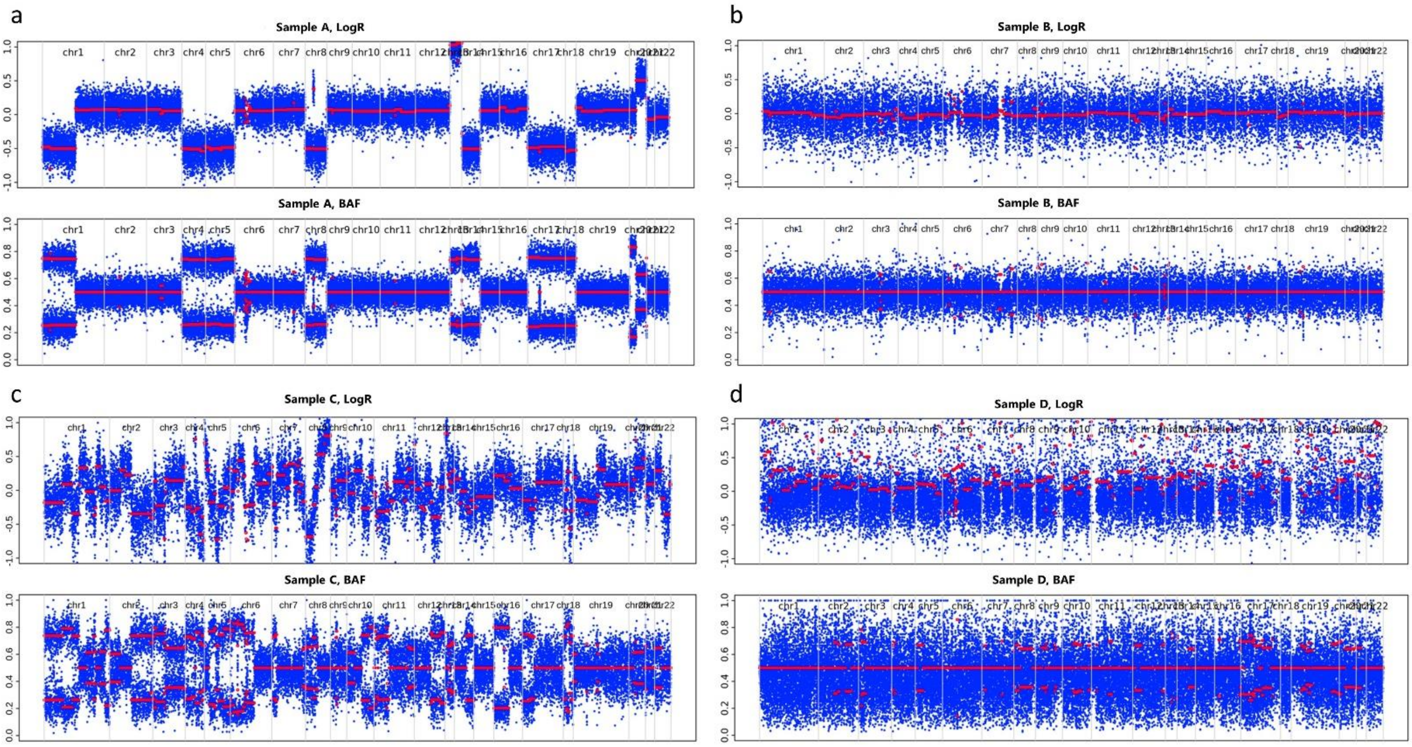

Figure S2: ASCAT plots of WES-derived input data. In each panel is shown matching logR (top) and BAF profiles (bottom) for a tumor sample. Chromosomes are marked on the X-axis. ***a****: A high quality tumor sample.* ***b****: A noisy sample, with no indications of CNAs, likely due to low tumor purity and high normal cell admixture.* ***c****: A noisy sample with hyper-fragmented LRRs, likely due to high levels of intra-tumor heterogeneity.* ***d****: A noisy tumor sample with abnormal BAFs and LRRs, possibly caused by low sequencing data quality or mismatched tumor and PBMC samples.*
